# Supplementary material for: The Potential of Plant-Based Bioactive Compounds on Inhibition of Aflatoxin B1 Biosynthesis and Down-regulation of aflR, aflM and aflP Genes
Source: Antibiotics (Basel). 2020 Oct 23;9(11):728. doi: 10.3390/antibiotics9110728 (PMC7690750; doi:10.3390/antibiotics9110728)
Supplement: Supplementary file 1 [file antibiotics-09-00728-s001.pdf]

antibiotics

MDPI

Article

Supplementary Materials: The Potential of Plant-Based Bioactive Compounds on Inhibition of Aflatoxin B1 Biosynthesis and Down-Regulation of *aflR*, *aflM* and *aflP* Genes

Nassim Safari [1,2,*], Mehran Mirabzadeh Ardakani [3], Roghayeh Hemmati [1], Alessia Parroni [2], Marzia Beccaccioli [2] and Massimo Reverberi [2,*]

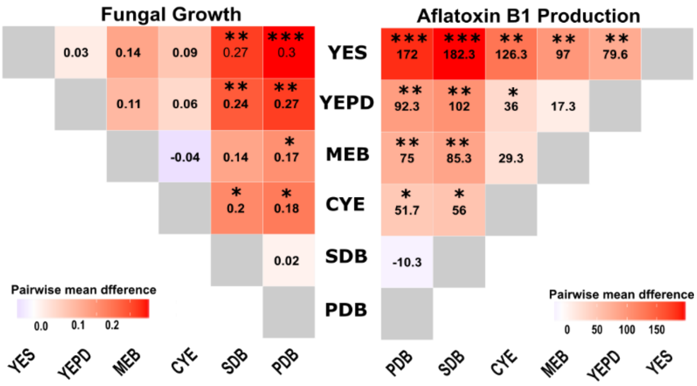

Figure S1. Selecting the optimal culture medium for *A. flavus* growth and aflatoxin B1 production. Pairwise comparisons of fungal growth/aflatoxin B1 production between six culture media: Potato Dextrose Broth (PDB), Sabouraud Dextrose Broth (SDB), Czapek Yeast Extract (CYE), Malt Extract Broth (MEB), Yeast Extract Peptone Dextrose (YEPD), and Yeast Extract Sucrose (YES) using Tukey HSD test. For example, the first row of the right table shows the differences of the mean- (across repetitions) aflatoxin B1 production between YES and all other mediums. Corresponding *p*-values obtained from Tukey HSD analysis (* $p < 0.05$, ** $p < 0.01$, *** $p < 0.001$). Our analysis showed that, compared to other culture media, the Yeast Extract Sucrose (YES) medium provides a highly significant condition for fungal growth and aflatoxin B1 production.

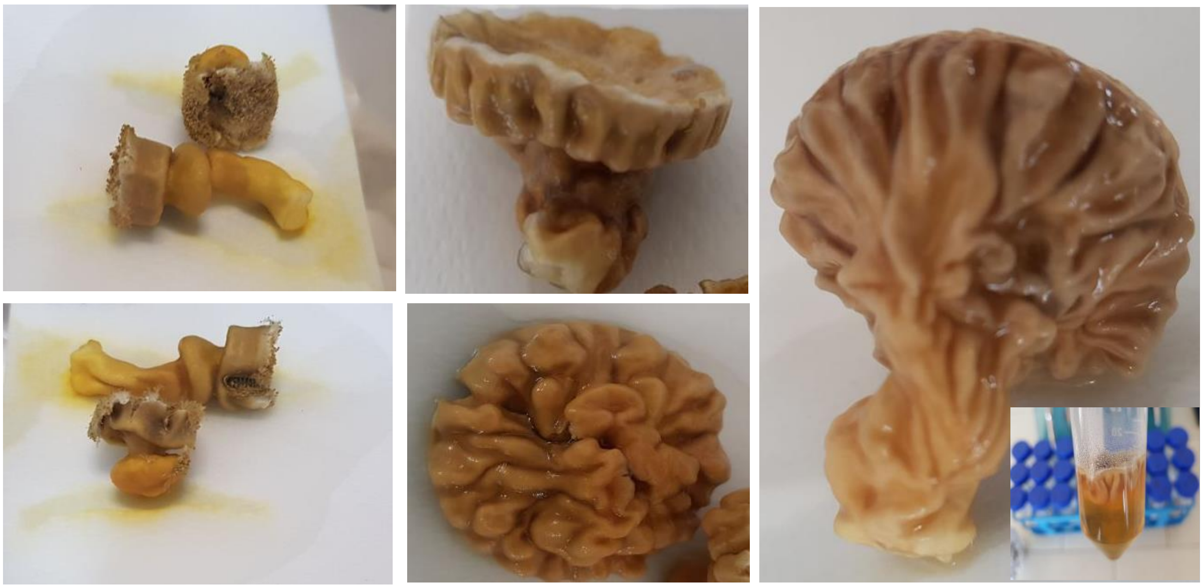

Figure S2. *A. flavus* strain NRRL 3357 Hyphal Growth treated with different doses of the plant extracts for 7 days at 28°C in Yeast extract-sucrose medium (YES).

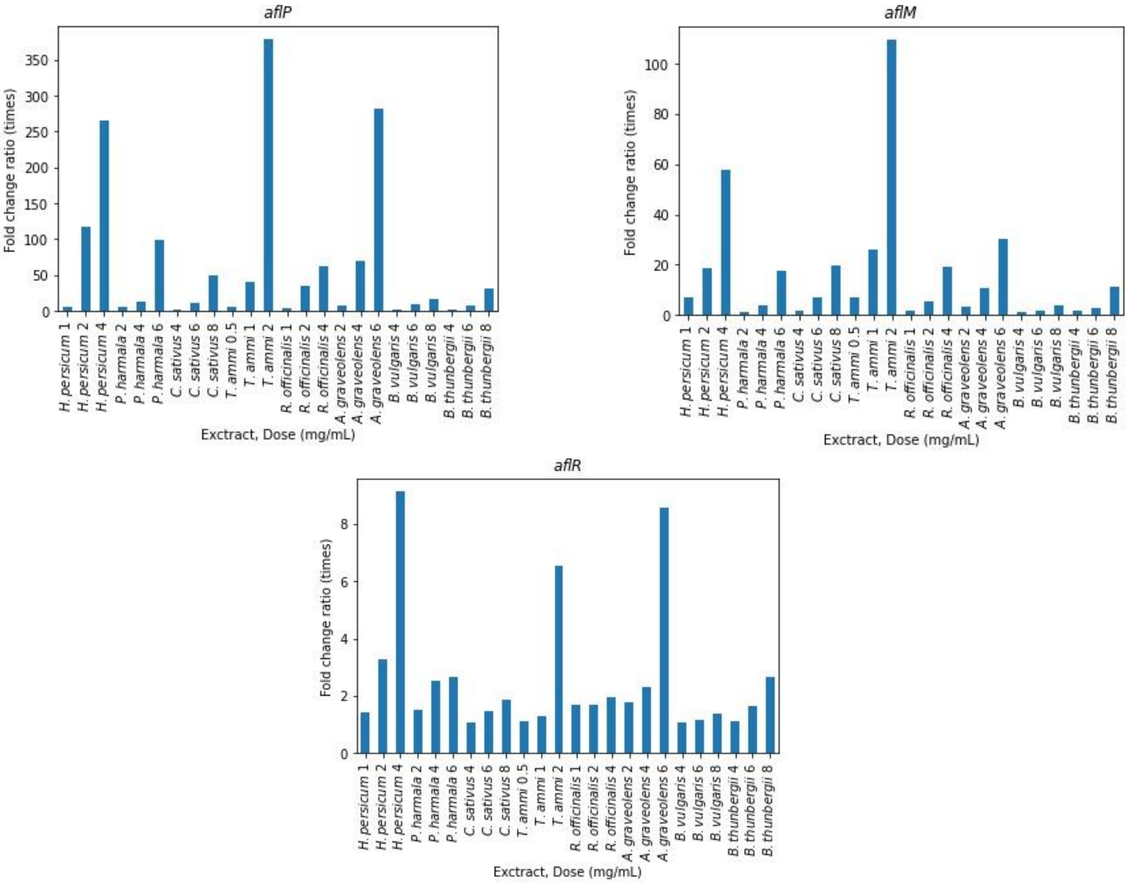

Figure S3. The fold-change reductions in expression levels compared to control conditions

Table S1. PCR condition for each gene

Gene	Initial denaturation		Denaturation		Annealing		Extension		Final extension	
	**Temp**	**Time**	**Temp**	**Time**	**Temp**	**Time**	**Temp**	**Time**	**Temp**	**Time**
aflR	95	10 min	95	15 s	60	30 s	72	2 min	72	5 min
aflM	95	10 min	95	15 s	60	30 s	72	2 min	72	5 min
aflP	95	10 min	95	15 s	57	30 s	72	2 min	72	5 min
tubulin beta	95	10 min	95	15 s	59	30 s	72	2 min	72	5 min
